# Setup errors analysis in iterative kV CBCT: A clinical study of cervical cancer treated with Volumetric Modulated Arc Therapy

**DOI:** 10.1002/acm2.14480

**Published:** 2024-08-09

**Authors:** Hui Xiao, Qiman Han, Shuhua Wei, Minghao Du, Xiuwen Deng, Nan Zhang, Chunxiao Li, Junjie Wang, Ang Qu, Ping Jiang

**Affiliations:** ^1^ Department of Radiation Oncology Peking University Third Hospital Beijing P. R. China

**Keywords:** cervical cancer, iterative kV cone beam CT (iCBCT), setup errors, volumetric modulated arc therapy (VMAT)

## Abstract

**Objective:**

This study aims to analyze setup errors in pelvic Volumetric Modulated Arc Therapy (VMAT) for patients with non‐surgical primary cervical cancer, utilizing the onboard iterative kV cone beam CT (iCBCT) imaging system on the Varian Halcyon 2.0 ring gantry structure accelerator to enhance radiotherapy precision.

**Method:**

We selected 132 cervical cancer patients who underwent VMAT with daily iCBCT imaging guidance. Before each treatment session, a registration method based on the bony structure was employed to acquire iCBCT images with the corresponding planning CT images. Following verification and adjustment of image registration results along the three axes (but not rotational), setup errors in the lateral (X‐axis), longitudinal (Y‐axis), and vertical (Z‐axis) directions were recorded for each patient. Subsequently, we analyzed 3642 iCBCT image setup errors.

**Results:**

The mean setup errors for the X, Y, and Z axes were 4.50 ± 3.79 mm, 6.08 ± 6.30 mm, and 1.48 ± 2.23 mm, respectively. Before correction with iCBCT, setup margins based on the Van Herk formula for the X, Y, and Z axes were 6.28, 12.52, and 3.26 mm, respectively. In individuals aged 60 years and older, setup errors in the X and Y axes were significantly larger than those in the younger group (*p* < 0.05). Additionally, there is no significant linear correlation between setup errors and treatment fraction numbers.

**Conclusion:**

Data analysis underscores the importance of precise Y‐axis setup for cervical cancer patients undergoing VMAT. Radiotherapy centers without daily iCBCT should appropriately extend the planning target volume (PTV) along the Y‐axis for cervical cancer patients receiving pelvic VMAT. Elderly patients exhibit significantly larger setup errors compared to younger counterparts. In conclusion, iCBCT‐guided radiotherapy is recommended for cervical cancer patients undergoing VMAT to improve setup precision.

## 1 INTRODUCTION

Cervical cancer, primarily caused by persistent infection with high‐risk types of Human Papillomavirus (HPV), is one of the most common malignancies worldwide and ranks as the fourth leading cause of cancer‐related deaths among women. In 2020, approximately 604 000 new cases of cervical cancer and 342 000 deaths were reported globally, posing a significant threat to women's health and safety.^1^, ^2^ Currently, the primary treatments for cervical cancer include surgery, radiotherapy, and chemotherapy, with radiotherapy playing an increasingly vital role in the disease's comprehensive management. According to the National Comprehensive Cancer Network (NCCN) guidelines (version 2.2024), patients with inoperable early‐stage, locally advanced, or recurrent and metastatic cervical cancer can receive radiotherapy for radical, palliative, or adjuvant treatments. With advancements in modern imaging technology, development of computerized treatment planning systems (TPS), and application of multi‐leaf collimator technology, radiotherapy has entered an era of unprecedented precision.^3^, ^4^ In clinical practice, it has been found that the accuracy of positioning is one of the key factors affecting the efficacy of radiotherapy. The ICRU Report 24 indicates that dosage deviations of ± 5% from the optimal dose for tumor targets can result in primary tumor control failure and increase the risk of local recurrence. With the advent of the three‐dimensional era, various technologies and devices have been developed to significantly improve the precision of radiation therapy. These include three‐dimensional conformal radiation therapy (3D‐CRT), stereotactic body radiotherapy (SBRT), volumetric modulated arc therapy (VMAT), and image‐guided radiation therapy (IGRT).^5^ One of the innovations in IGRT is kilovoltage cone‐beam CT (CBCT) that utilizes the Varian‐developed Acuros CTS algorithm.^6^ This algorithm simulates particle scatter transport processes to enhance image accuracy. iCBCT introduces iterative reconstruction algorithms, based on traditional CBCT, to improve overall image quality. iCBCT enables image acquisition before or during daily treatment sessions, supporting both online and offline verification and correction of clinical target volume (CTV) localization.^7^ This technology enhances treatment precision and minimizes adverse effects on organs at risk (OARs) by enabling accurate positioning of the tumor target and making timely adjustments.^8^

This study employed the Halcyon 2.0 iCBCT image‐guided system to scan cervical cancer patients before each treatment, acquire iCBCT images, align them with the planning CT images using the bone structure registration method, and make necessary corrections. The primary aims were to assess the accuracy of iCBCT in detecting the location of the tumor target by recording and analyzing patient setup errors, investigate the relationship between setup errors and patients' age, monitor the trend of setup errors throughout the treatment course, and perform an in‐depth analysis of the causes of these errors, thereby providing significant insights.

## 2 MATERIALS AND METHODS

### 2.1 General information

A total of 132 primary cervical cancer patients, who underwent pelvic VMAT using the Halcyon 2.0 iCBCT at Peking University Third Hospital, were sequentially selected from November 10, 2021 to June 29, 2023. None of these patients had previously undergone any gynecological surgery. The ages of patients ranged from 27 to 82 years, with an average age of 55.06 years. All patients received a standard regimen consisting of a single dose of 1.8−2.0 Gy, administered five times a week, totaling 25−28 sessions. A total of 3642 sets of iCBCT images were acquired for all patients before correction.

Inclusion criteria included the following: ① Patients with pathologically confirmed cervical cancer; ② Patients with no prior history of external pelvic radiation therapy; prior non‐pelvic radiation therapy was assessed on a case‐by‐case basis; ③ General physical condition (Karnofsky Performance Score) ≥70, indicating tolerance to radiotherapy; ④ The purpose and protocol of this study were explained to the patients and their families, and informed consent was obtained.

Exclusion criteria included the following: ① Bone marrow suppression; ② Severe dysfunction of major organs; ③ Acute or subacute pelvic inflammatory disease; ④ Active phase of a psychiatric disorder; ⑤ Severe infection.

### 2.2 Equipment and materials

Halcyon 2.0 linear accelerator onboard iCBCT (Varian Medical Systems, Palo Alto, USA), Eclipse TPS (ver.15.0, Varian Medical Systems, Palo Alto, USA), Philips Brilliance 16‐row 85 cm large aperture CT (Royal Philips, Netherlands), carbon fiber integrated plate, low‐temperature thermoplastic mesh membrane (specification: 56 cm × 46 cm), and feet fixation to stabilize the patient's legs (CIVCO Company, USA).

### 2.3 Methodology

#### 2.3.1 Preparation before CT simulation

Before CT simulation, patients underwent a detailed educational session on the treatment process and associated precautions. This session aimed to minimize psychological stress and enhance treatment compliance by covering topics such as expected outcomes, potential side effects, strategies for managing these effects, and maintaining quality of life during treatment. On the day of positioning, specialists instructed patients to empty their rectum and bladder. Subsequently, based on individual conditions, patients were advised to drink approximately 750−1000 mL of warm water mixed with 10 mL of iohexol to improve small intestine imaging. Patients were required to consume the water within 10 min and retain urine for an hour to ensure the bladder was adequately filled. A well‐distended bladder aids in maintaining consistent anatomy during radiation therapy, which is crucial for the accuracy of treatment delivery.

#### 2.3.2 Patient positioning and CT scanning

Patients were positioned in supine positions and immobilized with carbon fiber integrated plates and low‐temperature thermoplastic mesh membranes. Suitable headrests were placed under the patients' heads. Patients clasped their elbows, raising them above their foreheads, while straightening their legs on the feet fixation devices. The positioning involved laser light cross‐line alignment, with positioning lines drawn on the patients' skin to ensure consistent positioning during scanning and treatment. All patients underwent a contrast‐enhanced CT scan (Scanning parameters included a resolution of 512 × 512, voltage of 120 kV, and current of 325 mAs), except those with severe allergies or significant renal impairment. This approach enhanced the imaging of lymph nodes, blood vessels, and tumors, which was crucial for accurate target volume identification and critical structure avoidance. The scanning covered from the upper border of the 10th thoracic vertebra to 5 cm below the ischial tuberosity, with a 5 mm slice thickness. Following the completion of the scan, the acquired data were uploaded to the Eclipse TPS workstation in Digital Imaging and Communications in Medicine (DICOM) format. Subsequently, radiation oncologists meticulously outlined the CTV and OARs, ensuring treatment precision, while medical physicists developed the treatment plan, optimizing the dose distribution for effective tumor control with minimal exposure to surrounding healthy tissues.

#### 2.3.3 iCBCT imaging, image registration, and recording setup errors

**FIGURE 1:**
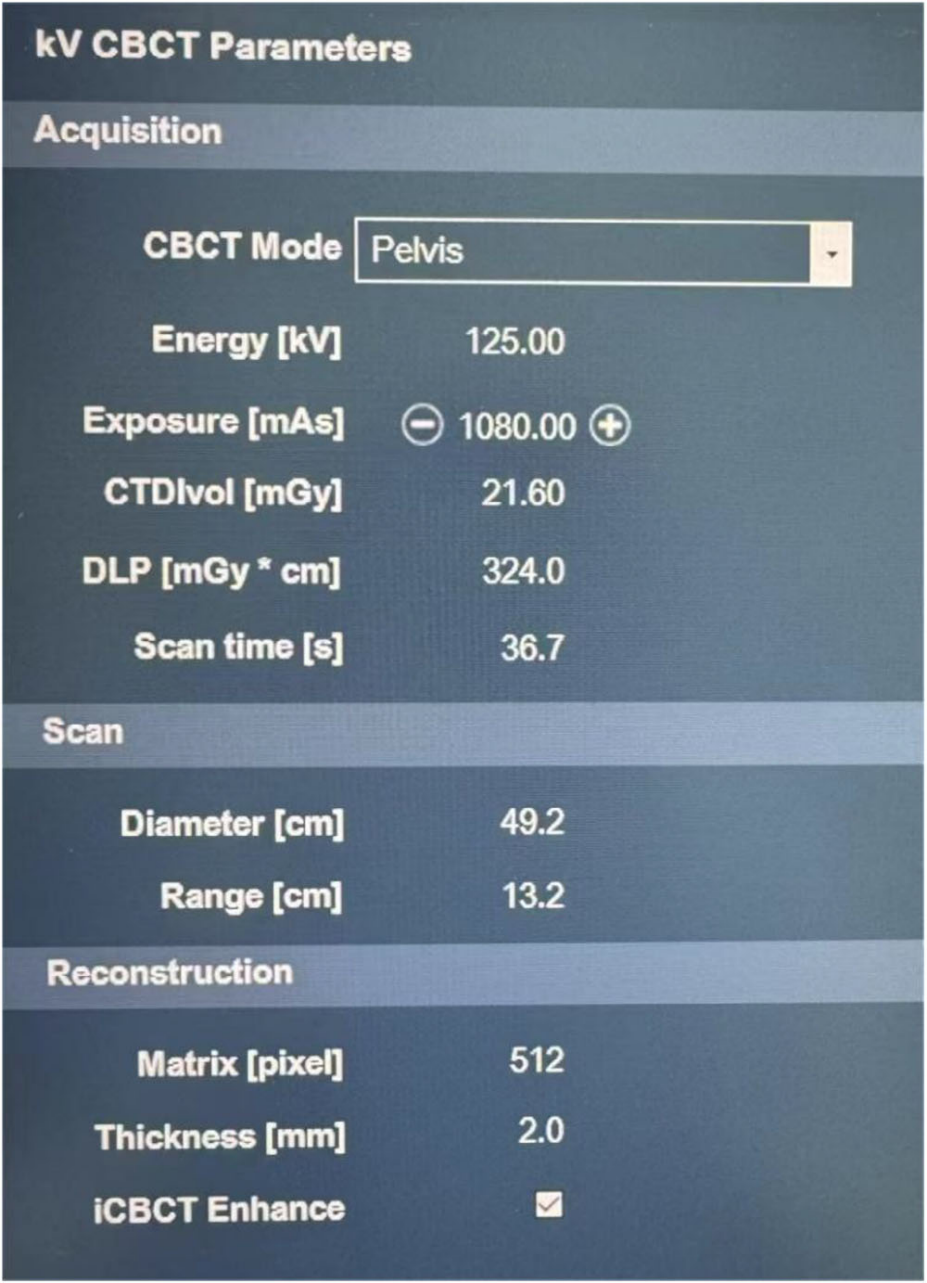
iCBCT scanning parameters.

**FIGURE 2:**
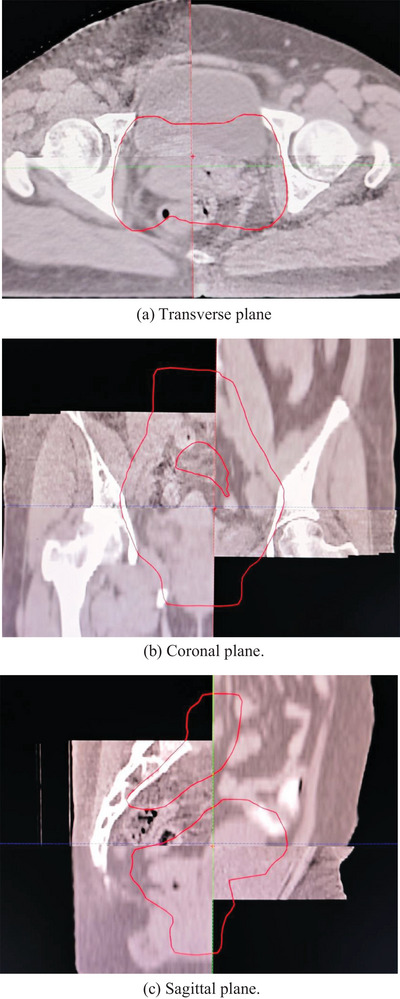
Three‐dimensional orientation of a cervical cancer patient undergoing iCBCT‐guided VMAT. The red area in the diagram represents PTV. In the three pictures, the upper left and lower right images are CT images, and the lower left and upper right images are iCBCT images. (a) Transverse plane. (b) Coronal plane. (c) Sagittal plane.

Before each treatment session, a single‐arc iCBCT scan was performed using the Varian linear accelerator. The scanning parameters of iCBCT were detailed in Figure 1. iCBCT images were acquired to serve as floating images for image registration, selecting an appropriate 3D matching box that defined the tumor target. This 3D volume was then matched to the corresponding volume in the planning CT images. Matching could be conducted manually or automatically, using options like bony alignment and grayscale alignment. Given the pelvis's rigid nature and clear visibility, bony structure registration targeted the pubic symphysis, sacroiliac joints, and bilateral iliac crests as priorities, with automatic alignment often attempted initially. iCBCT images were compared with the planning CT images, adjusting positions and angles to optimize overlap of the bony structures in the CTV and adjacent organs across axial, coronal, and sagittal planes, as shown in Figure 2. If CTV registration was unsatisfactory, manual alignment and fine‐tuning were performed based on the tumor's characteristics. If manual alignment proved unsatisfactory, repositioning was required. If repositioning failed to resolve the issue, a new planning CT scan and a revised radiation therapy plan were required, followed by the repetition of the above steps. After physicians and therapists verified the image registration results and made adjustments along the three axes (but not rotational), the inter‐fractional translation distance from the iCBCT image to the planning CT was defined as the setup error. This includes the displacement distance of automatic bone registration and manual fine‐tuning. Setup errors were recorded in three dimensions: Lateral movements to the left were recorded as negative values, and to the right as positive values. Longitudinal movements inward were recorded as positive, and outward as negative. Vertical movements upward were recorded as positive, and downward as negative. For error correction, our radiation therapy center implemented online corrections for deviations less than 5 mm. For deviations exceeding 5 mm but less than 20 mm, we implemented online corrections and adjusted the positioning lines on the patient's body surface. Deviations exceeding 20 mm required therapists to enter the treatment room to reposition the patient, redraw alignment lines on the patient's body surface, and repeat the iCBCT scan. During this process, therapists typically needed about 90 s to position the patient. The iCBCT scan lasted approximately 36.7 s. The process of registering bony structures from iCBCT images with planning CT images, along with manual fine‐tuning, lasted about 30−60 s. Consequently, the total preparation time before treatment was approximately 3 min.

#### 2.3.4 Statistical methods

The statistical analyses were performed using SPSS version 26.0. To accurately analyze the magnitude of setup errors, all error values were converted to absolute values. Such transformation was essential to eliminate the possibility of error value compensation caused by the varying directions of errors, thereby guaranteeing an accurate representation of the error magnitudes. The setup errors were quantified and presented as mean ± standard deviation (SD), facilitating an examination of the distribution patterns within the measurement data. The data underwent tests for normality and homogeneity of variance. If the data conformed to a normal distribution and exhibited homogeneity of variance, an independent sample *t*‐test was performed. For data not adhering to a normal distribution or displaying unequal variances, non‐parametric tests (the Mann‐Whitney *U* test) were utilized. This allowed for a more accurate evaluation of statistical differences in placement errors between groups. A *p*‐value < 0.05 was considered statistically significant.

## 3 RESULTS

### 3.1 Analysis of setup error data and calculation of theoretical margin expansion values before iCBCT correction

**FIGURE 3:**
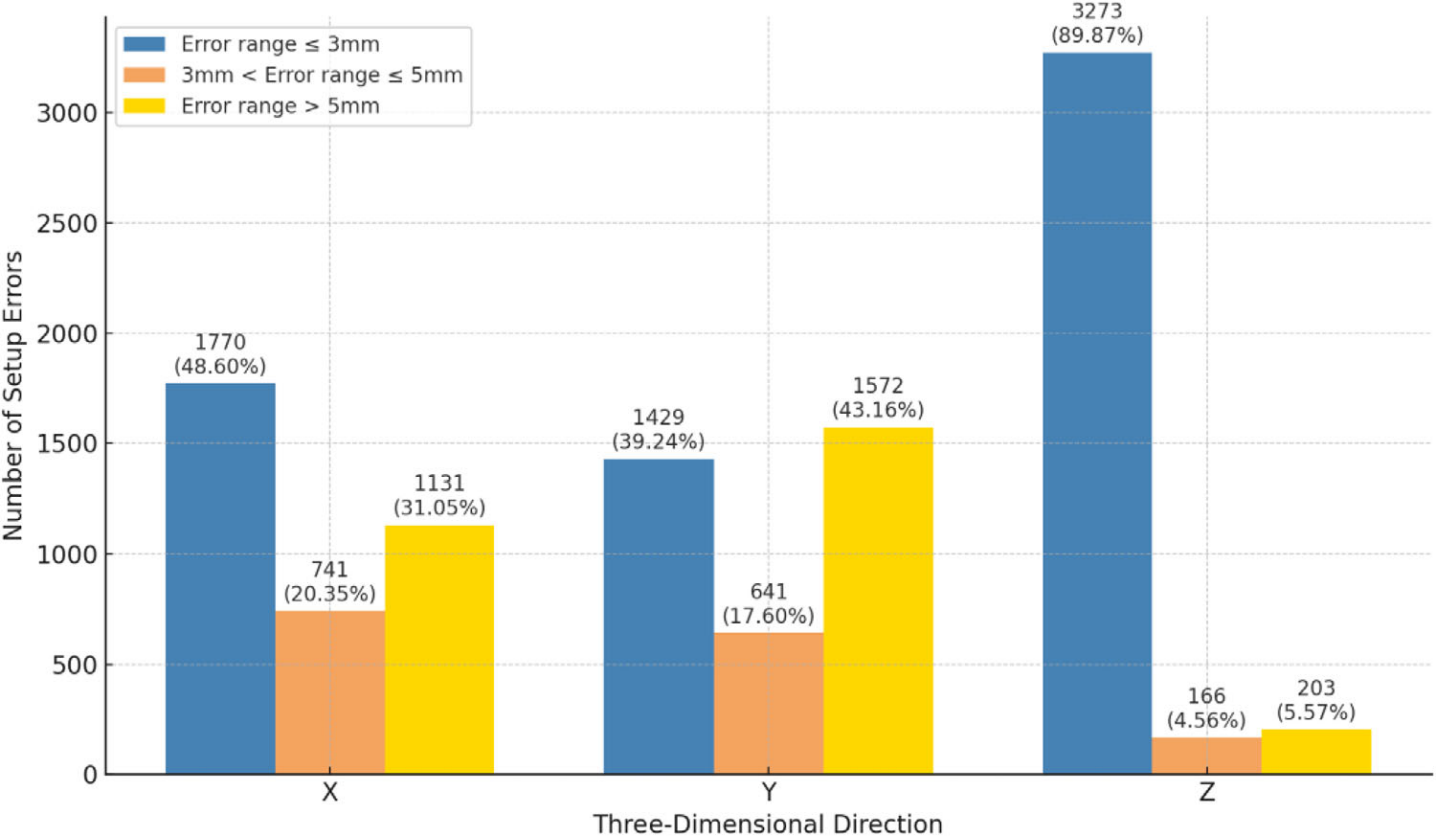
Distribution of three‐dimensional setup errors.

A total of 3642 registration image sets were analyzed and calculated, revealing mean setup errors for the X‐axis, Y‐axis, and Z‐axis were 4.50 ± 3.79 mm, 6.08 ± 6.30 mm, and 1.48 ± 2.23 mm, respectively. Errors on the X‐axis ranged from >3 mm to ≤5 mm, accounting for 20.35% (741 sets), while errors exceeding 5 mm accounted for 31.05% (1131 sets). Similarly, errors on the Y‐axis ranged from >3 mm to ≤5 mm, accounting for 17.60% (641 sets), while errors exceeding 5 mm accounted for 43.16% (1572 sets), as shown in Figure 3. Maximum setup errors were recorded at 41 mm for the X‐axis, 96 mm for the Y‐axis, and 22 mm for the Z‐axis, as shown in Table 1. These results underscored the importance of further optimizing Y‐axis positioning accuracy.

**TABLE 1:** Analysis results of the setup errors from 3642 iCBCT registrations for 132 cervical cancer patients (Units: Millimeters).

Variable	X axes	Y axes	Z axes
Minimum value	0	0	0
Maximum value	41	96	22
Mean	4.50	6.08	1.48
SD	3.79	6.30	2.23
*∑*	1.52	3.52	0.70
*σ*	3.54	5.31	2.16
*M*	6.28	12.52	3.26

In the intricate process of radiotherapy, treatment precision is significantly influenced by a myriad of factors, categorized into two primary types: systematic and random errors. Systematic error is a regular deviation, usually resulting from medical equipment parameter settings, linear accelerator calibration, laser alignment errors, target delineation deviations, treatment plan transmission, and more. Such errors can be reduced by improving equipment accuracy and optimizing treatment processes. Additionally, random error is an unpredictable deviation, referring to the variability in the patient's position during treatment, such as daily weight changes and involuntary movements of the CTV, including slight differences in treatment postures and physiological movements like breathing. This type of error is challenging to minimize through standardized procedures. According to research by Van Herk^9^, ^10^ to reduce the impact of setup errors and ensure that the minimum cumulative dose to 90% of the CTV reaches at least 95% of the prescribed dose, a population‐based formula for calculating the planning target volume (PTV) margin expansion was derived: *M*_PTV_ = 2.5 *Σ* + 0.7 *σ*. The *Σ* (population systematic error) is defined as the standard deviation of individual systematic errors, with each individual systematic error being the mean setup error for a patient. The *σ* (population random error) is defined as the root mean square of individual random errors, with each individual random error being the standard deviation of the setup error for a patient. If iCBCT image errors are not corrected before therapy, calculations based on 2.5 *Σ* + 0.7 *σ* result in theoretical expansion margins of 6.28, 12.52, and 3.26 mm for the X, Y, and Z axes, respectively, as shown in Table 1.

### 3.2 Relationship between setup errors and age

Patients were divided into two age‐based groups: those under 60 years in the younger group (Group A), comprising 86 patients with 2367 sets of iCBCT image data, and those aged 60 and above in the elderly group (Group B), comprising 46 patients with 1275 sets of iCBCT image data. Setup errors were statistically analyzed using SPSS version 26.0 to compare the discrepancies between Group A and Group B. Following a single‐sample Kolmogorov‐Smirnov normality test, which revealed that the data did not follow a normal distribution, the non‐parametric Mann‐Whitney *U* test was employed to compare the medians of the two independent samples, expressed as interquartile ranges (IQR). In Table 2, the *U*‐value quantifies the magnitude of difference between two independent samples; a *U*‐value approaching its minimum implies a significant disparity, whereas higher values suggest lesser differences. The *Z*‐value, which standardizes the *U*‐value, assesses the statistical significance of these differences. A larger absolute *Z*‐value indicates greater significance: positive values suggest the first sample is larger, while negative values suggest it is smaller. An absolute *Z*‐value greater than approximately 1.96 is considered statistically significant. A *p‐*value less than 0.05 indicates a significant difference between the two samples. In the younger group, the inter‐fraction setup errors along the X, Y, and Z axes were 3 (2, 6), 5 (2, 8), and 1 (0, 2) mm, respectively. For the elderly group, these errors were 4 (2, 7), 5 (2, 9), and 1 (0, 2) mm, respectively. Statistically significant differences were observed between the two groups in the errors along the X and Y axes (X‐axis: *Z* = −4.629, *p* < 0.001; Y‐axis: *Z* = −2.297, *p* = 0.022), with the elderly group showing larger setup errors than the younger group. The Z‐axis differences were not statistically significant (*p* > 0.05). Detailed data are presented in Table 2.

**TABLE 2:** Comparison of setup errors across different age groups (Units: Millimeters).

Variable	X axes	Y axes	Z axes
Group A	3.00	5.00	1.00
(2367 sets)	(2.00–6.00)	(2.00–8.00)	(0.00–2.00)
Group B	4.00	5.00	1.00
(1275 sets)	(2.00–7.00)	(2.00–9.00)	(0.00–2.00)
*U*	1 369 628.000	1 439 686.500	1 504 232.000
*Z*	−4.629	−2.297	−0.165
*p*	0.000	0.022	0.869

### 3.3 Relationship between setup errors and treatment fraction number

**FIGURE 4:**
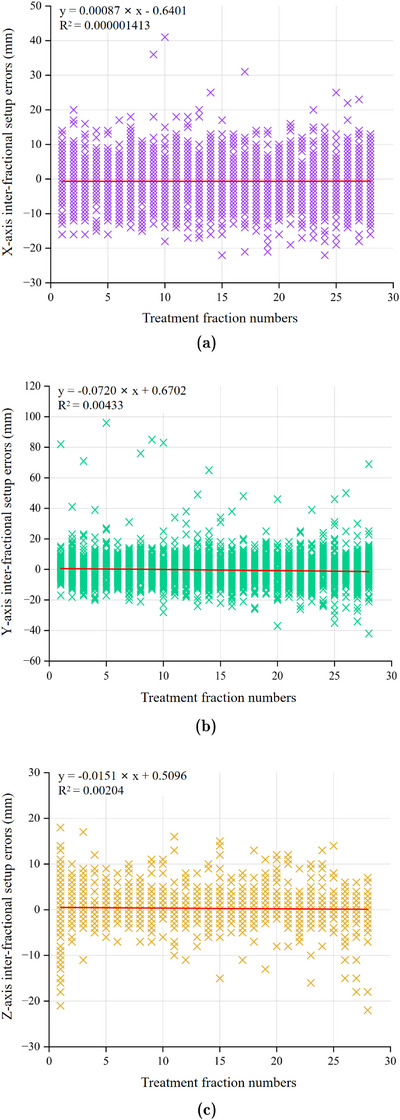
Linear regression analysis of three‐dimensional directional setup errors and treatment fraction numbers. (a) The relationship between X‐axis inter‐fractional setup errors and treatment fraction numbers. (b) The relationship between Y‐axis inter‐fractional setup errors and treatment fraction numbers. (c) The relationship between Z‐axis inter‐fractional setup errors and treatment fraction numbers.

To systematically evaluate whether setup errors in the X, Y, and Z axes varied with increasing treatment fraction numbers, this study employed linear regression analysis to quantify the relationship between these errors and treatment fraction numbers and to assess the statistical significance of the observed trends. The linear regression equation was defined as *y* = *mx* + *b*, where “m” represented the slope, indicating the rate of change in the dependent variable for each unit increase in the independent variable. The intercept “*b*” denoted the expected value of the dependent variable when the independent variable was zero. *R*‐squared, the coefficient of determination, quantified the proportion of variance in the dependent variable that was explained by the independent variable. Figure 4a depicted the linear regression equation *y* = 0.00087 × *x* − 0.6401, showing a negligible linear relationship between treatment fraction numbers and X‐axis setup errors. The exceptionally low slope suggested that treatment fraction numbers had minimal impact on X‐axis errors. Figure 4b presented the linear regression equation *y* = −0.0720 × *x* + 0.6702. The intercept *b* was 0.6702. The negative slope suggested that as treatment fraction numbers increased, Y‐axis errors initially exhibited a slight decrease, followed by a slight increase. Figure 4c depicted the linear regression equation *y* = −0.0151 × *x* + 0.5096, indicating an insignificant linear relationship between treatment fraction numbers and Z‐axis setup errors. The minimal slope suggested that increased treatment fraction numbers had a negligible effects on Z‐axis errors. Overall, the influence of treatment fraction numbers on setup errors across the X, Y, and Z axes was minimal, with insignificant variations observed in the errors across these axes.

## 4 DISCUSSION

The study utilized a registration method based on bony structures, with manual adjustments if necessary, to record inter‐fractional setup errors before treatment. It aimed to analyze the overall distribution of 3642 setup errors, the relationship between age and setup errors, and the correlation between treatment fraction numbers and setup errors. Based on extensive data, the Van Herk formula was applied to calculate theoretical expansion margins of 6.28, 12.52, and 3.26 mm for the X, Y, and Z axes, respectively. This observation is in line with the findings of Laursen et al.^11^ and Patni et al.,^12^ who similarly noted the largest discrepancy in Y‐axis positioning. At our center, cervical cancer patients undergoing pelvic VMAT receive daily iCBCT guidance. Despite relatively large setup errors on the Y‐axis, after correction, the PTV is still derived by extending the CTV by 5 mm. However, for cervical cancer patients receiving external beam radiation therapy (EBRT) without daily iCBCT guidance, it is recommended to appropriately expand the PTV along the Y‐axis.

Regarding the relationship between age and setup errors, particular attention should be paid to the positioning of elderly individuals aged 60 and above. This observation is consistent with clinical experiences, which show that elderly patients typically exhibit lower compliance, more deformable skin, reduced glomerular filtration rate, bladder control difficulties, stronger intestinal reactions, and a general decline in physical functions, all contributing to increased setup errors. Radiotherapy plays a crucial role and is widely utilized in the treatment of cancer among elderly patients. The Geriatric Oncology Society recommends incorporating the unique needs of elderly cancer patients into both clinical practice and radiotherapy research.^13^ A nuanced understanding of how age influences setup errors enables radiotherapists to devise more accurate, patient‐specific positioning strategies, ultimately enhancing the accuracy of treatment while minimizing radiation exposure.

Cervical cancer patients typically undergo a course of VMAT lasting more than a month. During this treatment period, various changes in the patient's physical condition and tumor biology can occur. These changes significantly impact the management and precision of treatment. Den et al.^14^ observed that the mean *M*_PTV_ along the Y‐axis was higher in weeks 4−6 of radiotherapy compared to weeks 1−3, suggesting an increasing trend in setup errors with treatment progression. We performed linear regression analysis to assess the relationship between setup errors in the X, Y, and Z axes and treatment fraction numbers. Our findings indicated no significant linear correlation between these variables.

In clinical practice, we have observed that setup errors are influenced by a variety of factors. Systematic errors can lead to shifts in the tumor volume, while random errors can blur the dose at the tumor target's margins. Understanding the causes of these setup errors and reducing them is essential for patient benefits during radiotherapy. Observations indicate that, in addition to the previously detailed analysis, several key factors significantly contribute to the setup errors in radiotherapy, including: ① Tumor volume changes: Cervical cancer treatment is predominantly comprehensive, with some patients' tumors responding positively to radiation therapy and shrinking rapidly. Herrera et al.^15^ conducted a study involving 10 patients with cervical cancer. The findings revealed that during radiotherapy, clinical target volume—simultaneous integrated boost decreased by 31%–70%. The average displacements of the tumor center were 12.5 mm in the cranio‐caudal direction, 9 mm in the anteroposterior direction, and 3 mm in the lateral direction. As a result of organ motion and tumor shrinkage, the conformity between the actual radiation dose received by the tumor target and the treatment plan rapidly decreased. In two patients, the dose delivered to the tumor target was less than 95% of the prescribed dose, leading to an overdose in adjacent normal organs. Lee et al.^16^ observed that during the combined treatment of EBRT, cisplatin chemotherapy, and high‐dose‐rate brachytherapy (HDR‐BT), the volume of cervical cancer tumors rapidly decreases. The study calculated that the time for 50% tumor volume regression was 21 days, occurring after receiving 30.8 Gy. Additionally, Van de Bunt et al.^17^ reported an average 46% reduction (range 6.1%–100%) in primary total tumor target after approximately 30 Gy of EBRT. Changes in tumor target can lead to deviations in image registration when comparing treatment images with the planning CT images. Therefore, we advocate for regular assessments of disease progression. For patients experiencing rapid tumor shrinkage, iCBCT can serve as a monitoring tool. In such cases, re‐simulation and re‐planning with CT are crucial for maintaining treatment accuracy. ② The complexity of the organs in the pelvic cavity: Women's pelvic organs include the uterus, bladder, rectum, etc. The displacement of these organs significantly affects the dose distribution within the tumor target, complicating the determination of the PTV boundary.^18^, ^19^, ^20^ During cervical cancer radiotherapy, Mahantshetty et al.^21^ found that uterine motion resulted in average displacements of 4.5–5.3 mm in all directions. In the mid‐cervical region, movements averaged between 4.5 and 5.5 mm. Notably, shifts in the superior direction of the uterine fundus were significantly larger, with an average displacement of 12.1 mm. Beadle et al.^22^ reported that the average cervical volume before and after 45 Gy of external irradiation was 97.0 and 31.9 cc, respectively, indicating an average volume reduction of 62.3%. Research has shown that changes in bladder volume impact the CTV; specifically, every 10 cm^3^ decrease in bladder filling resulted in an 8 mm shift in the cervical canal and an 18 mm shift in the base, cumulatively causing an approximate cervical shift of 3 mm.^23^ Variable bladder filling can lead to uterine apex movement ranging from 0 to 65 mm anteroposteriorly and more than 40 mm vertically.^24^ Rectal filling induced an average anterior‐posterior shift of the cervix by 4.1 mm, with minimal effect on the uterus's lateral movement.^25^ ③ Respiratory motion: The respiratory movements of patients, notably abdominal breathing, induce vertical shifts in surface markers. Effectively managing these movements is crucial for maintaining the accuracy of radiation delivery, as it ensures that radiation is precisely targeted to the tumor while minimizing exposure to surrounding healthy tissues.^26^ While iCBCT facilitates tumor location verification in VMAT treatments, 3D‐CBCT lacks real‐time tracking of tumor movement trajectories, potentially compromising tumor target precision during radiotherapy. However, 4D‐CBCT tracks the tumor's movement trajectory with remarkable accuracy, enabling precise control over the radiation field's range and the conformity of CTV during radiotherapy.^27^ ④ Body shape changes and weight factors: Many patients undergo radiotherapy and chemotherapy simultaneously. Factors such as adverse reactions to chemotherapy and fear of the disease, which can lead to weight loss, may cause a mismatch between body shape and low‐temperature thermoplastic mesh membrane. Consequently, it may be necessary to adjust the initially planned radiotherapy area to ensure that radiation precisely targets the intended area. Additionally, studies have indicated that obesity significantly influences the occurrence of setup errors during the treatment of female pelvic tumors. Lin et al.^28^ observed that for women of normal or slightly above‐average weight, a planned margin extension of 7–10 mm was typically adequate, even without IGRT. However, for patients with moderate to severe obesity, this margin may be inadequate, necessitating wider expansion margins. ⑤ Equipment factors: To ensure the accuracy of radiation therapy and patient safety, it is essential to conduct regular precision checks and maintenance of equipment, including radiation therapy devices, image‐guided systems, and three‐dimensional positioning lasers. Additionally, before treatment, it is crucial to inspect long‐used immobilization devices for adaptability, stability, and signs of wear, softening, or deformation. These devices require careful monitoring for changes in their physical state, with replacements made as necessary to maintain treatment precision. ⑥ Radiotherapy technician factors: The accuracy of patient positioning is directly influenced by the professional skills, ethical conduct, and meticulous attention to detail of radiotherapy technicians. Given the evolving nature of radiotherapy technology and treatment protocols, it is highly recommended to organize regular training sessions dedicated to the enhancement of professional skills and the dissemination of knowledge updates. Furthermore, the assessment of technicians' abilities and operational skill improvements should be conducted through annual evaluations, fostering an environment of continuous learning and professional development. Ensuring adherence to the most current standard operating procedures is critical, and these procedures should be regularly updated to mirror the latest clinical guidelines and technological advancements in the field. The adoption of a dual‐technician approach for patient positioning is advised as a strategic measure to minimize errors. These approaches improve the accuracy of patient positioning, significantly reducing the potential for errors and raising the quality of therapy received by patients undergoing radiotherapy.

## 5 CONCLUSIONS

In conclusion, setup errors in VMAT for cervical cancer patients show significant variability. For radiotherapy centers that administer VMAT for cervical cancer without daily iCBCT guidance, it is advised to appropriately expand PTV based on specific circumstances. During the treatment process, it is critical to focus on the precise positioning of elderly patients and to adapt to dynamic changes, such as tumor shrinkage and organ movement, to ensure the accuracy of radiotherapy. Recommendations for minimizing setup errors include refining positioning systems, strengthening patient education, optimizing bladder and rectal function management, closely monitoring changes in tumor area, conducting regular equipment checks, providing extensive training for medical staff, employing advanced image‐guided technologies, and customizing treatment plans to individual patient conditions, among others. Moreover, future research should explore the use of artificial intelligence (AI) and machine learning (ML) algorithms in analyzing patterns from historical setup errors data to predict and automatically correct potential deviations in real‐time. Establishing comprehensive databases and patient registries will be pivotal in systematically collecting data on therapeutic efficacy and adverse reactions to radiotherapy, facilitating ongoing improvements in treatment protocols and iCBCT precision.

## AUTHOR CONTRIBUTIONS

**Hui Xiao**: Conceptualization; methodology; software; validation; formal analysis; investigation; writing—original draft preparation; writing—review and editing. **Qiman Han**: Conceptualization; methodology; investigation; visualization; writing—review and editing. **Shuhua Wei**: Formal analysis. **Minghao Du**: Data curation. **Xiuwen Deng**: Software. **Nan Zhang**: Data curation. **Chunxiao Li**: Supervision. **Junjie Wang**: Resources; supervision. **Ang Qu**: Validation; resources; visualization; supervision; project administration. **Ping Jiang**: Resources; project administration funding acquisition.

## CONFLICT OF INTEREST STATEMENT

The authors declare no conflicts of interest..

## ETHICS APPROVAL AND CONSENT TO PARTICIPATE

All procedures performed in this study were in accordance with the ethical standards of the institutional research committee. Written informed consent was provided by all patients.

## Data Availability

All data can be obtained by contacting the corresponding author.
